# Prediction of respiratory failure risk in patients with pneumonia in the ICU using ensemble learning models

**DOI:** 10.1371/journal.pone.0291711

**Published:** 2023-09-21

**Authors:** Guanqi Lyu, Masaharu Nakayama

**Affiliations:** Department of Medical Informatics, Tohoku University Graduate School of Medicine, Miyagi, Japan; The First Hospital of Jilin University, CHINA

## Abstract

The aim of this study was to develop early prediction models for respiratory failure risk in patients with severe pneumonia using four ensemble learning algorithms: LightGBM, XGBoost, CatBoost, and random forest, and to compare the predictive performance of each model. In this study, we used the eICU Collaborative Research Database (eICU-CRD) for sample extraction, built a respiratory failure risk prediction model for patients with severe pneumonia based on four ensemble learning algorithms, and developed compact models corresponding to the four complete models to improve clinical practicality. The average area under receiver operating curve (AUROC) of the models on the test sets after ten random divisions of the dataset and the average accuracy at the best threshold were used as the evaluation metrics of the model performance. Finally, feature importance and Shapley additive explanation values were introduced to improve the interpretability of the model. A total of 1676 patients with pneumonia were analyzed in this study, of whom 297 developed respiratory failure one hour after admission to the intensive care unit (ICU). Both complete and compact CatBoost models had the highest average AUROC (0.858 and 0.857, respectively). The average accuracies at the best threshold were 75.19% and 77.33%, respectively. According to the feature importance bars and summary plot of the predictor variables, activetx (indicates whether the patient received active treatment), standard deviation of prothrombin time-international normalized ratio, Glasgow Coma Scale verbal score, age, and minimum oxygen saturation and respiratory rate were important. Compared with other ensemble learning models, the complete and compact CatBoost models have significantly higher average area under the curve values on the 10 randomly divided test sets. Additionally, the standard deviation (SD) of the compact CatBoost model is relatively small (SD:0.050), indicating that the performance of the compact CatBoost model is stable among these four ensemble learning models. The machine learning predictive models built in this study will help in early prediction and intervention of respiratory failure risk in patients with pneumonia in the ICU.

## Introduction

Respiratory failure is a common complication in patients hospitalized for pneumonia [1]. Bacterial, aspiration, viral, and fungal pneumonia can lead to respiratory failure [2–5], resulting in serious complications. Patients with respiratory failure bear high medical costs; the average length of hospitalization for patients diagnosed with respiratory failure in the United States in 2017 was 10.5 days and the average cost of treatment at hospital was $158,443 [6], and in the United States alone, the government spends more than $5 billion annually on acute respiratory failure [7]. In addition, respiratory failure poses a serious threat to patients’ lives, especially in critically ill patients admitted to the intensive care unit (ICU). The in-hospital mortality rate of critically ill patients with respiratory failure is 33–37% [1], and the treatment process for respiratory failure is also difficult and challenging [8]. Furthermore, in addition to leading to patient death, patients with respiratory failure are at risk for hypoxia, which may induce delirium, long-term cognitive impairment and hippocampal atrophy. [9–13]. Respiratory failure is a great burden in the public health system [6, 14, 15]; it causes suffering and harm to patients with pneumonia. Therefore, early prediction of respiratory failure risk in patients with severe pneumonia admitted to the ICU is important.

Mining and analyzing clinical big data, such as electronic health records, through machine learning techniques has brought great convenience to the medical field. The importance of disease prediction analysis is increasing [16–18]. Machine learning techniques are also applicable for respiratory failure [19] However, studies related to the early prediction of respiratory failure in patients with pneumonia using ensemble learning algorithms, are limited.

In recent years, ensemble learning algorithms have demonstrated relatively good performances in machine learning competitions and research using machine learning. These algorithms fit structured data better and have a lower computational cost and robust performance in handling of missing values and outliers. In addition, their output directly provides the order of feature importance, which significantly enhances the interpretability of the model [20, 21]. In this study, four ensemble learning algorithms, LightGBM; XGBoost; CatBoost; and random forest, were used to build early prediction models for respiratory failure risk in patients with severe pneumonia. The risk of developing respiratory failure in patients with severe pneumonia one hour after ICU admission was predicted by using data before and within one hour after ICU admission. In our study, we predicted the risk as early as possible and compared the predictive effectiveness of the four models. Additionally, to improve the utility and interpretability of the models, a feature selection method based on the order of feature importance was used to develop compact models corresponding to the four complete models. Finally, feature importance and Shapley additive explanations (SHAP) were introduced to improve the interpretability of the models.

## Materials and methods

### Data sources

The data used in this study were obtained from the eICU Collaborative Research Database (eICU-CRD), which is a large multicenter critical care database provided by Philips Healthcare in collaboration with the MIT Computational Physiology Laboratory. It contains data on more than 200,000 critical care patients in more than 200 healthcare facilities in multiple states across the United States between 2014 and 2015. The patient data in eICU-CRD has been de-identified and no personal information can be accessed; it includes data on vital signs, care plan documentation, diagnostic information, treatment information, laboratory results, and demographics. These data can support the functioning of applications such as machine-learning models, and the development of clinical decision tools [22]. The data used in this study were extracted from a public database and data has been de-identified; therefore, informed patient consent or ethical approval from the Institutional Review Board (IRB) was not required. All procedures were performed in accordance with the ethical standards of the 1964 Declaration of Helsinki and its later amendments or comparable standards. Access to the data was granted to the researchers upon completion of CITI Data or Specimens Only Research training. We got approval to extract data from the eICU-CRD. This study was ethically approved by the Massachusetts Institute of Technology Affiliates (Record ID:53432794).

### Study population and endpoints of interest

#### Study population

First, patients admitted to the ICU for bacterial, viral, fungal, aspiration and parasitic pneumonia, and pneumonia labeled as other types, as per the eICU-CRD were included. For patients with multiple ICU admissions, only the records of the first ICU admission were selected. To ensure the reliability of the results, we excluded patients who did not have a diagnostic record during the period of ICU admission. Patients were not excluded based on age, as it may also influence the risk of developing respiratory failure.

#### Endpoints of interest

In this study, the following three criteria were used to determine the occurrence of respiratory failure in patients with severe pneumonia in the ICU: (I) documented diagnosis of respiratory failure one hour after admission to the ICU. The textual record of the diagnoses included the following options: respiratory failure, acute respiratory failure, and ICD-9 codes 518.81, 518.84, and 518.5; (II) arterial partial pressure of oxygen (PaO_2_) of <60 mmHg one hour after admission to the ICU; (III) arterial partial pressure of carbon dioxide (PaCO_2_) >45 mmHg one hour after admission to the ICU [1, 23, 24].

Among the three definition criteria mentioned above, the relationship between definitions (I), (II), and (III) is an intersection and the relationship between definitions (II) and (III) is a union. The predictive accuracy of the occurrence of respiratory failure in patients with severe pneumonia in the ICU is jointly ensured by these three criteria as errors may exist in diagnostic records.

### Predictor variables

In this study, medical history records, laboratory results, periodic and aperiodic vital signs data, medication records, demographic data, and APACHE III calculation variables of patients were extracted before and within 1 h of ICU admission.

### Data pre-processing

As most of the variables were missing, retaining only complete variables or variables with few missing values would have resulted in a significant loss of information. Therefore, variables with less than 80% missing values were retained to maintain the maximum amount of original patient information. The multivariate imputations by chained equations (MICE) missing value filling method was used to fill in the missing values [25]. A total of 1784 predictor variables were included.

In this study, we did not perform outlier processing on data, such as laboratory results and vital signs, because these data may vary in real situations depending on patient conditions and certain values with special significance recorded by doctors. Therefore, if outlier processing is performed, useful features may be lost.

In the eICU-CRD, a patient’s age is marked as ’ >89 ’ if it is greater than 89 years. Such data cannot be processed using the ensemble-learning algorithms. Therefore, in this study, the age of patients older than 89 years was replaced as 90 years.

### Complete prediction models

A total of four ensemble learning algorithms (LightGBM, XGBoost, CatBoost, and random forest) were used to build prediction models for respiratory failure risk in patients with severe pneumonia in the ICU. Optuna [26], which is an automated hyperparameter-tuning framework based on the Bayesian optimization algorithm, was used to optimize hyperparameter combinations by selecting those most likely to achieve better results at each step, thereby ensuring more efficient and faster tuning.

To more accurately represent the generalization ability of the models, the dataset was subjected to the following steps: first, randomly divide the whole dataset into training and test sets at a ratio of 9:1; second, perform 10-fold cross validation on the training set to operate the tuning of hyperparameters; third, use the same training set to build prediction models and calculate the corresponding average area under receiver operating curves (AUROCs) on the test set; and lastly, loop this process 10 times to obtain the average AUROC values of the models on 10 different test sets.

Finally, the average AUROC of each model on the test sets was obtained, and the average prediction accuracy under the best threshold of each model was calculated.

### Compact prediction models

As 1784 predictor variables are required in the complete prediction models, their practical use and model interpretability in the clinic is difficult. Therefore, in this study, corresponding compact prediction models for respiratory failure risk based on each complete prediction model were developed.

Feature selection was performed on the predictor variables based on feature importance. The predictor variables of each model were ranked according to the order of feature importance from highest to lowest. Prior to feature selection, the dataset was first randomly divided into training set and test set at a ratio of 9:1, and then the training set was divided at a ratio of 9:1 again to obtain the new training set and the independent validation set. Next, the models were re-instantiated and the best combination of hyperparameters corresponding to each model obtained from the previous Optuna hyperparameter tuning was passed in.

Starting from the first variable in a training set, only one variable was selected at a time for model training and prediction, and the AUROC of the model on the validation set at this time was recorded. This entire process was incorporated in one round. If the AUROC of the validation set obtained after training and testing the model with the current variable was greater than that of the previous validation set, the variable selected in this round was retained. If the AUROC of the validation set obtained after training and testing the model with the current variables was less than or equal to that of the previous validation set, the variables selected in this round were excluded until the number of selected variables reached 20 or until all 1784 variables were tested.

Each compact model was then evaluated for hyperparameter optimization and generalization performance, using the same methods as those used for the complete prediction models.

Finally, four compact models were compared. The SHAP summary plot and feature importance bar graph were plotted for the compact prediction model with the highest average AUROC to improve the model interpretability.

### Statistical analysis

For predictor variables with multiple measurements, the maximum, minimum, mean, and standard deviations were calculated separately. For the analysis of baseline characteristics, Categorical variables were presented as percentages, and continuous variables were presented as mean ± standard deviation for normally distributed continuous variables or median (interquartile range) for non-normally distributed continuous variables. Categorical variables were compared using the chi-square test. Normally distributed continuous variables were compared between the groups using Student’s t-test. The Shapiro-Wilk test was performed to determine whether the continuous variables were normally distributed. In addition, non-normally distributed continuous variables were compared using the Mann–Whitney U test, and differences were considered statistically significant when the p-value was less than 0.001.

The database used in this study is PostgreSQL 15.1. Data pre-processing, comparison of baseline characteristics, and model building for this study were done in Jupyter Notebook 6.4.8 in Anaconda Navigator 2.3.2, and the programming language used was Python 3.9.12.

## Results

### Baseline characteristics

A total of 1676 patients with pneumonia were finally included in this study. Of these, 297 patients developed respiratory failure one hour after admission to the ICU, and the incidence of respiratory failure in patients with pneumonia in the ICU was 17.7%. The groups of patients with pneumonia who developed and did not develop respiratory failure were named the positive and negative groups, respectively. As the number of predictor variables was too large, only the representative laboratory results, periodic vital signs, non-periodic vital signs, demographic data and Glasgow Coma Scale (GCS) scores were used for statistical analysis.

The baseline characteristics of the patients with severe pneumonia are shown in Table 1. Patients in the positive group had lower serum calcium levels, faster respiratory and heart rates, younger age, higher weight and body mass index (BMI), and lower GCS scores than those in the negative group.

**Table 1:** Baseline characteristics.

Variables	Total (*N* = 1676)	Negative group (*N* = 1379)	Positive group (*N* = 297)	*P*_value
**Demographic data**				
Age (year)	69.00 (56.00–80.00)	70.00 (57.00–81.00)	63.00 (53.00–75.00)	<0.001
Female (%)	46.3	45.7	49.2	0.306
Admission height (m)	1.69 (1.60–1.78)	1.69 (1.60–1.78)	1.68 (1.62–1.78)	0.928
Admission weight (kg)	77.00 (63.00–91.00)	75.50 (62.65–89.00)	81.70 (65.00–101.60)	<0.001
BMI	26.50 (22.81–31.87)	26.06 (22.66–31.07)	28.22 (23.38–34.85)	<0.001
**Periodic vital signs**				
Respiration (BPM*^1^)	23.38 (20.00–25.82)	23.29 (19.73–25.33)	23.62 (21.18–28.64)	<0.001
SaO2 (%)	95.91 (94.55–97.91)	95.99 (94.81–97.91)	95.74 (93.73–97.67)	0.003
Heartrate (BPM*^2^)	93.19 (81.50–103.94)	93.01 (80.82–103.00)	93.90 (85.64–109.36)	<0.001
**Aperiodic vital signs**				
Noninvasive systolic (mmHg)	120.55 (106.00–131.85)	120.50 (105.50–131.25)	120.71 (109.33–135.50)	0.121
Noninvasive mean (mmHg)	81.66 (72.00–89.14)	81.56 (71.75–88.49)	81.96 (72.50–92.00)	0.308
Noninvasive diastolic (mmHg)	66.17 (57.74–73.00)	66.00 (57.50–71.75)	66.59 (59.25–76.00)	0.020
**Lab results**				
WBC (K/mcL)	13.30 (10.20–14.50)	13.30 (10.10–14.76)	13.30 (11.00–13.78)	0.928
RBC (M/mcL)	4.01 (3.70–4.24)	4.01 (3.70–4.27)	4.00 (3.70–4.08)	0.206
Hgb (g/dL)	11.80 (10.80–12.60)	11.80 (10.80–12.70)	11.80 (10.85–11.96)	0.240
Hct (%)	35.65 (33.20–38.00)	35.75 (33.20–38.50)	35.55 (33.06–35.99)	0.004
MCV (fL)	90.18 (88.40–92.11)	90.18 (88.20–92.50)	90.17 (89.00–91.00)	0.377
MCH (%)	29.74 (29.10–30.54)	29.73 (29.09–30.60)	29.76 (29.38–30.10)	0.826
MCHC (g/dL)	32.86 (32.45–33.30)	32.86(32.40–33.30)	32.86 (32.60–33.00)	0.626
RDW (%)	15.51 (14.48–15.92)	15.49 (14.40–15.95)	15.54 (14.93–15.87)	0.030
Platelets (K/mcL)	241.42 (189.00–258.00)	241.52 (188.00–265.50)	241.22 (196.00–243.86)	0.220
Total bilirubin (mg/dL)	0.86 (0.60–0.89)	0.85 (0.60–0.89)	0.87 (0.60–0.90)	0.031
AST (Units/L)	44.00 (27.00–56.68)	43.00 (26.00–54.99)	49.66 (30.00–61.83)	<0.001
ALT (Units/L)	37.43 (23.00–47.17)	36.34 (23.00–46.30)	42.61 (24.00–51.68)	<0.001
Glucose (mg/dL)	147.35 (115.38–147.53)	147.32 (115.00–147.53)	147.40 (118.00–147.53)	0.071
Bicarbonate (mmol/L)	24.99 (24.00–26.00)	24.99 (24.00–26.00)	24.96 (23.50–25.47)	0.289
BUN (mg/dL)	24.88 (17.00–29.10)	24.94 (17.00–29.56)	24.78 (19.00–28.44)	0.796
Creatinine (mg/dL)	1.44 (0.90–1.60)	1.41 (0.89–1.60)	1.49 (0.94–1.60)	0.221
Sodium (mmol/L)	136.32 (135.00–138.30)	136.31 (135.00–138.50)	136.36 (135.88–138.00)	0.867
Potassium (mmol/L)	4.12 (3.80–4.24)	4.11 (3.80–4.30)	4.13 (3.90–4.17)	0.125
Chloride (mmol/L)	100.25 (98.00–102.76)	100.27 (98.00–103.00)	100.17 (99.00–101.00)	0.538
Calcium (mg/dL)	8.79 (8.60–8.90)	8.79 (8.60–9.00)	8.78 (8.50–8.81)	<0.001
BNP (pg/mL)	376.00 (151.25–1467.25)	355.00 (135.40–1435.20)	470.00 (196.15–1742.00)	<0.001
**GCS scores**				
Motor	6.00 (6.00–6.00)	6.00 (6.00–6.00)	6.00 (5.00–6.00)	<0.001
Eyes	4.00 (4.00–4.00)	4.00 (4.00–4.00)	4.00 (2.00–4.00)	<0.001
Verbal	5.00 (4.00–5.00)	5.00 (4.00–5.00)	4.00 (1.00–5.00)	<0.001

Non-normally distributed continues values in **Total** column, **Negative group** column and **Positive group** column are presented as Median (IQR); IQR, interquartile range.

Abbreviations: BPM*^1^, Breaths Per Minute, BPM*^2^, Beats Per Minute; SaO2, Arterial Oxygen Saturation; WBC, White Blood Cell; K/mcL, Thousand per microliter; RBC, Red Blood Cell; M/mcL, Million per microliter; Hgb, Hemoglobin; Hct, Hematocrit; MCV, Mean Corpuscular Volume; MCH, Mean Corpuscular Hemoglobin; MCHC, Mean Corpuscular Hemoglobin Concentration; RDW, Red Cell Distribution Width; AST, Aspartate Aminotransferase; ALT, Alanine Aminotransferase; BUN, Blood Urea Nitrogen; BNP, Brain Natriuretic Peptide.

### Model predictive performance

#### Complete prediction models

The average AUROC after prediction and the average accuracy at the best threshold for each complete model using the optimal combination of parameters on 10 different test sets are shown in Table 2. The complete CatBoost model had the highest average AUROC after prediction on 10 different test sets (AUROC:0.858, SD:0.050), indicating that this model had strong discriminative power for prediction of respiratory failure risk in patients with pneumonia in the ICU.

**Table 2:** Predictive performance of each complete model.

Model	AUROC	Average accuracy
LightGBM	0.831±0.045	77.03%
XGBoost	0.836±0.054	75.60%
CatBoost	0.858±0.050	75.19%
RandomForest	0.832±0.041	75.19%

AUROC are presented as $\bar{x}\pm sd$. sd, standard deviation.

LightGBM, Light Gradient Boosting Machine; XGBoost, eXtreme Gradient Boosting; CatBoost, Categorical Boosting; AUROC, area under receiver operating characteristic curve.

**Fig 1:**
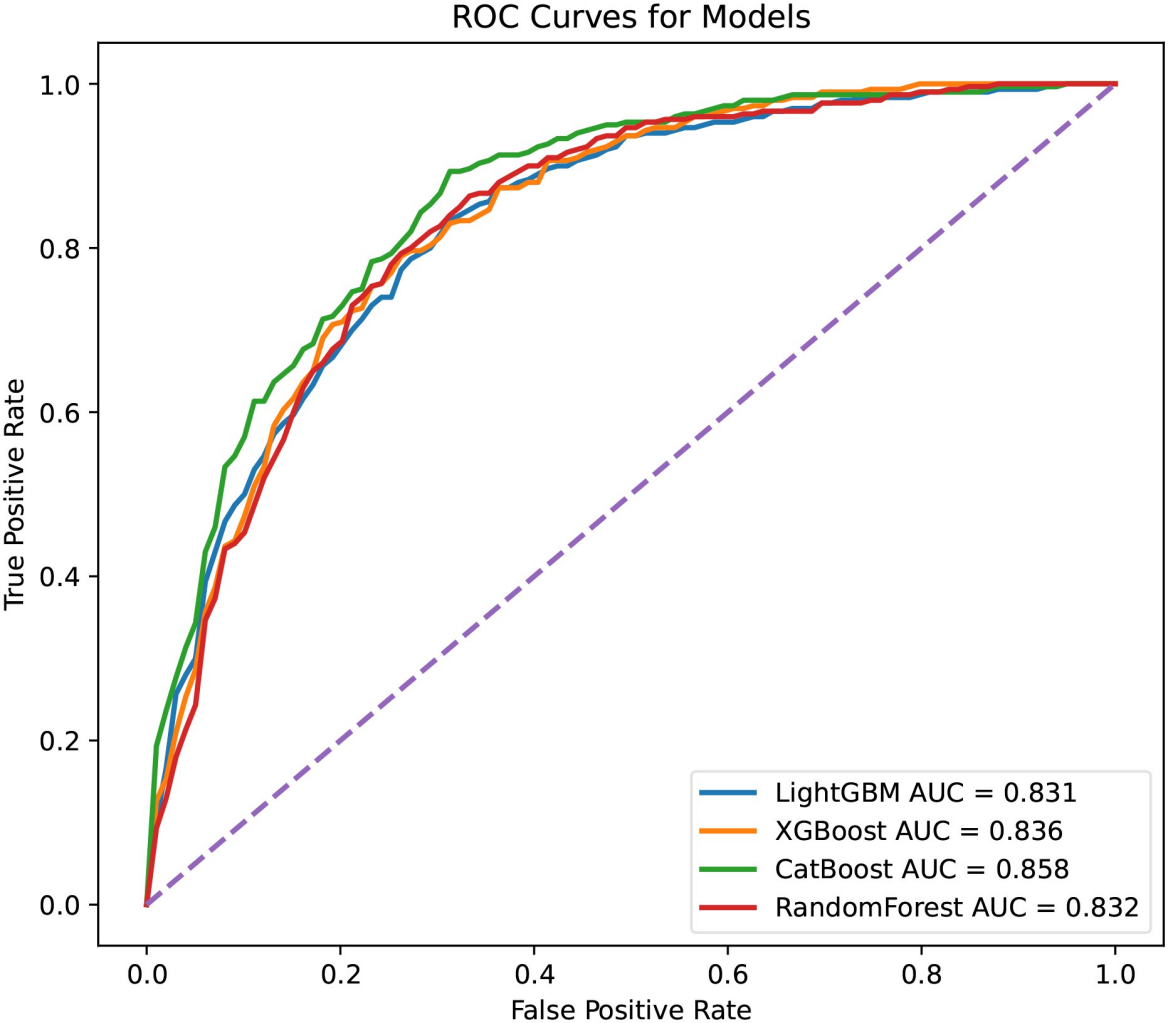
Receiver operating curves of complete models.

The ROC curves for each complete model are shown in Fig 1. XGBoost and RandomForest models had a slightly low average AUROC than the CatBoost model, while the LightGBM model had the lowest average AUROC. The optimal hyperparameter combinations for each complete model are listed in S1 Table.

#### Compact prediction model

The average AUROC, average accuracy at the best threshold, and number of predictor variables used for each compact model using the optimal hyperparameter combination after prediction on 10 different test sets are listed in Table 3. Among them, the compact CatBoost model had the highest average AUROC (AUROC:0.857, SD:0.050) after testing on 10 different test sets.

**Table 3:** Prediction performance of each compact model.

Model	Number of features	AUROC	Average accuracy
LightGBM	18	0.820±0.052	76.26%
XGBoost	20	0.824±0.058	77.09%
CatBoost	20	0.857±0.050	77.33%
RandomForest	20	0.838±0.045	73.76%

AUROC is presented as $\bar{x}\pm sd$. sd, standard deviation.

**Fig 2:**
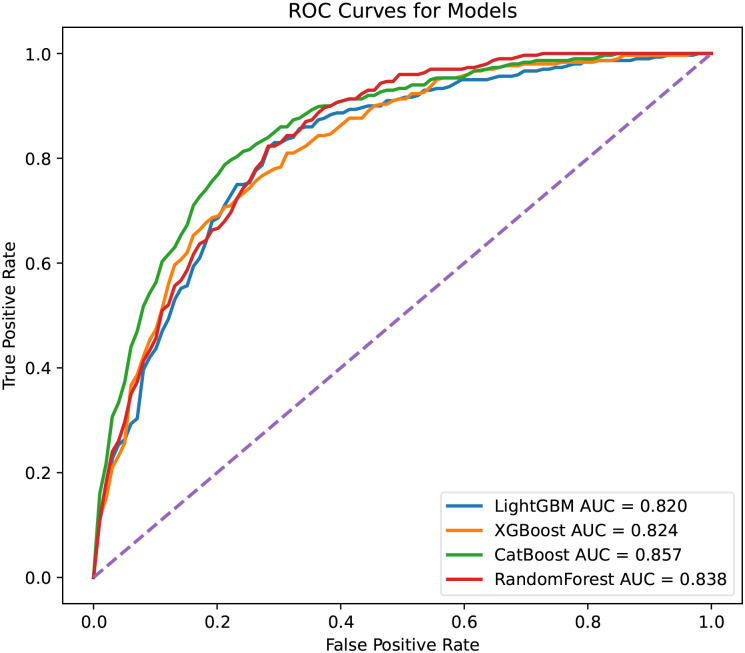
Receiver operating curves of compact models.

The ROC curves for all compact models are shown in Fig 2. The prediction performances of LightGBM and XGBoost models were decreased compared with that of the complete prediction model. After feature selection, the CatBoost model with only 20 predictor variables had a similar AUROC to the previous complete model and maintained the highest average AUROC among the four compact ensemble learning models. The optimal hyperparameter combinations for each compact model are listed in S2 Table.

### Model interpretation

#### Feature importance

**Fig 3:**
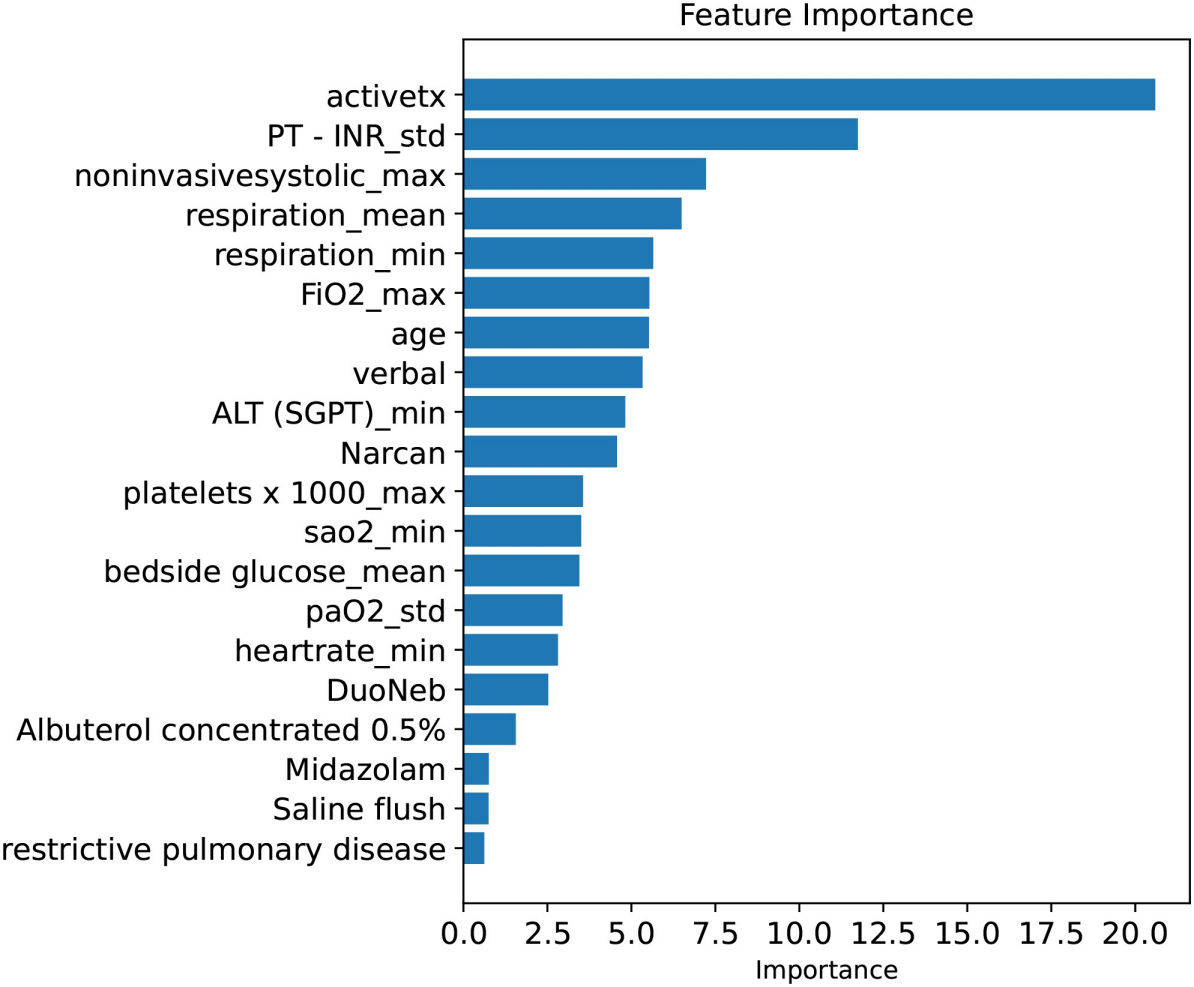
Feature importance of CatBoost compact model.

The importance of the 20 predictor variables provided by the CatBoost algorithm with the highest AUROC among the four compact prediction models is shown in Fig 3. The top-ranked predictor variables were activetx, PT-INR_std, noninvasivesystolic_max, respiration_mean, respiration_min, FiO2_max, age, and verbal, which were dominated by laboratory results, vital signs, and APACHE III calculation variables. The feature importance bar shows the features that have the greatest impact on the model; however, it cannot explain the relationship between the features and predicted outcomes. Thus, the gap values can be explored.

#### SHAP summary plot

**Fig 4:**
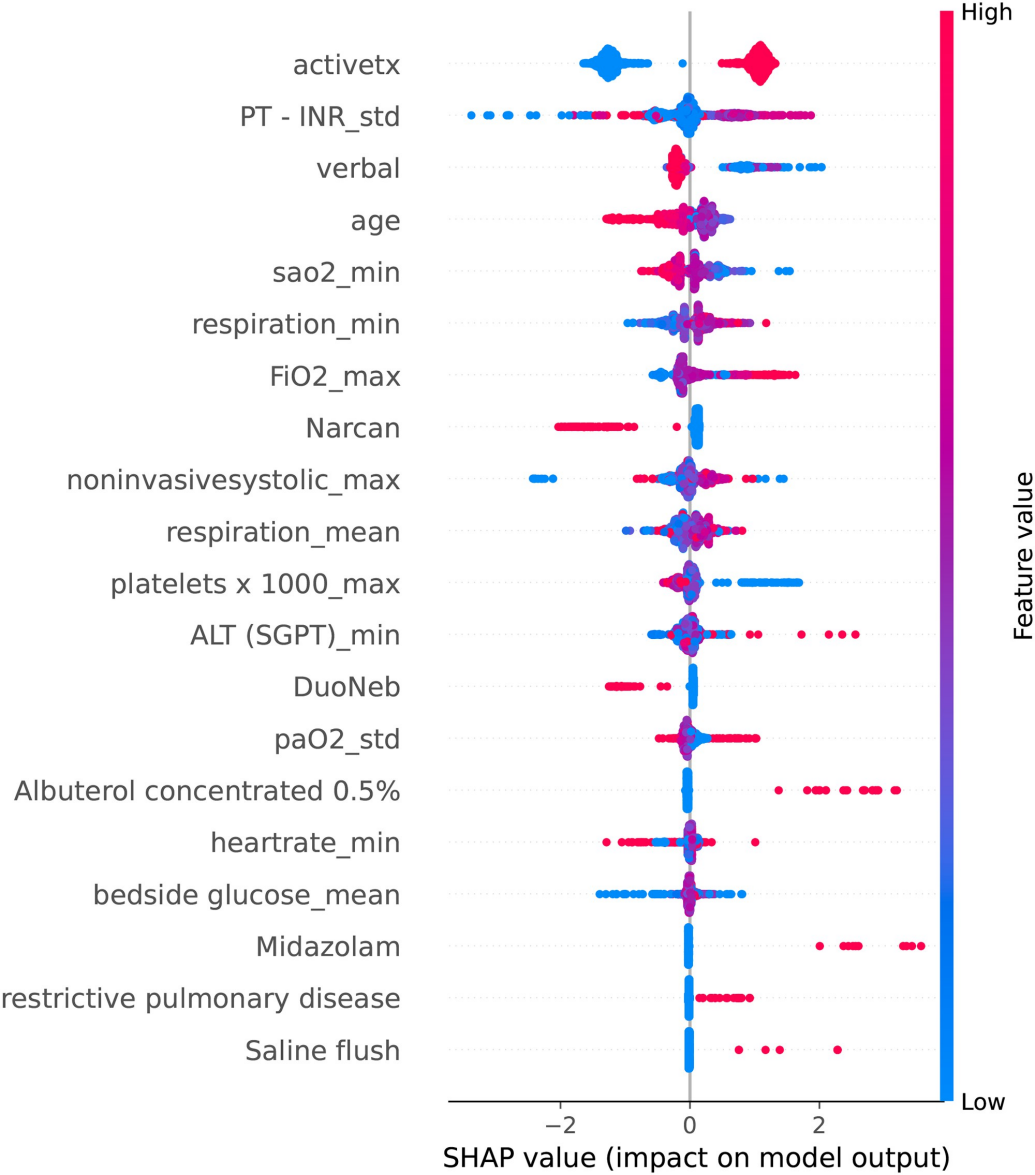
SHAP summary plot of CatBoost compact model.

The SHAP summary plot is shown in Fig 4, which represents the relative importance of each feature based on the SHAP value. Each row in the plot represents a predictor variable, and each point represents a sample clustered in the vertical direction. Red and blue color indicate high and low values of the predictor variables, respectively. The x-axis shows the effects of the predictor variables on the prediction results. The right and left directions indicate positive and negative effects of the predictor variable, respectively, on the prediction result.

The predictor variables Narcan, DuoNeb, 0.5% albuterol, Midazolam, Saline Flush, and restrictive pulmonary disease in Fig 4 are categorical variables with values of 0 or 1, indicating the presence or absence of drug use and history of restrictive pulmonary disease. A value of 1 indicated use of the drug by patient or presence of disease history, while a value of 0 indicated that the patient did not use the drug or absence of disease history.

As shown in Figs 3 and 4, activetx is the most important feature for early prediction of respiratory failure risk in patients with severe pneumonia. In the eICU-CRD, activetx is a categorical variable with values of 0 and 1, indicating whether the patient has received active treatment. Therefore, the summary plot showed that patients who received active treatment had a higher risk of respiratory failure. Moreover, a higher standard deviation of prothrombin time-international normalized ratio (PT-INR) test, that is, higher degree of variability of PT-INR, and lower GCS verbal score and oxygen saturation are risk factors for the development of respiratory failure in patients with severe pneumonia.

## Discussion

There is a lack of reliable tools for early prediction of respiratory failure risk in patients with severe pneumonia [19]. In this study, we used the eICU-CRD, a large multicenter critical care database provided by Philips Healthcare in collaboration with the MIT Computational Physiology Laboratory, to develop prediction models for respiratory failure risk in patients with severe pneumonia based on four ensemble learning algorithms. To improve the utility of the prediction models, a feature selection method based on feature importance was used to develop a compact model corresponding to each model.

Our study showed that ensemble learning algorithms can accurately predict respiratory failure risk in patients with severe pneumonia. Moreover, in this study, only laboratory results, vital signs data, medication records, APACHE III calculation variables, demographic data, and history of disease before and within one hour of admission to the ICU were used as predictor variables; therefore, the respiratory failure risk could be predicted in a relatively short period after admission of patients with pneumonia to the ICU.

In this study, the cross-validation and hyper-parameter tuning process was rational and scientific. CatBoost showed the highest average AUROC in both the complete models and the compact models. (AUC of complete CatBoost model: 0.858±0.050, AUC of compact CatBoost model: 0.857±0.050). The compact models of XGBoost, LightGBM, and RandomForest, showed more significant performance degradation compared with their respective complete models. However, the performance of CatBoost was almost unchanged, with an AUC decrease of only 0.001, which is much more stable compared with the other three models. The CatBoost model has two main advantages over other ensemble learning algorithms. First, it can directly process categorical variables without prior coding, which speeds up model fitting. Second, it is more powerful in preventing model overfitting, thereby, significantly improving the generalization ability of the model [27].

In this study, we found that activetx (indicates whether active treatment received by the patient) was the most important predictor variable. This study also showed that patients with pneumonia who received active treatment were more likely to experience respiratory failure. Pneumonia patients with an activetx of 1 received 163 more types of treatment than those with an activetx of 0, indicating difference in treatment between the two patient groups. The treatment modalities mainly included mechanical ventilation; use of multiple powerful antibiotics such as vancomycin, ampicillin, and azithromycin; use of multiple drugs for deep fungal infections such as fluconazole, itraconazole, and voriconazole; and use of noradrenaline for the treatment of shock. The application of these treatment modalities indicate serious bacterial or fungal infection and critical condition of the patient. As the official documentation of the eICU-CRD does not define the conditions required for activetx value to be 1, based on the above description, we believe that the activetx value of 1 may indicate that the patient was receiving more important and critical treatments, and in this study, these treatments are indicative of a more complex condition with a high risk of developing respiratory failure. Thus, whether a patient with pneumonia receives more specific and frequent active treatments against the disease that is currently most predominant may indicate that patient’s severity. In addition, we found that the standard deviation of the PT-INR, GCS verbal score, platelet count, oxygen saturation and respiratory rate were important predictor variables.

This study showed that patients are more likely to develop respiratory failure if their platelet count is low. This is also consistent with findings of previous research. Rodrigues et al. showed that 145 coronavirus disease (COVID-19) patients with moderate to severe acute respiratory failure had 15.1% of hemorrhagic events during ICU admission. Coagulation disorders were more prevalent in patients with high levels of critical illness [28]. Another study by Wolny et al. also showed that in critically ill patients with COVID-19, platelet counts were significantly lower and platelet function was impaired compared with mildly ill patients [29]. Some possible explanations for the specific causes are as follows. First, platelets have a crucial role in the body’s immune system, and platelets have various receptors on their surface that interact with immune cells to fight off external infections; thus, a lowered platelet count can lead to damage of the immune system, which can worsen pneumonia and lead to respiratory failure [29]. Second, platelets are mainly produced from megakaryocytes, which are abundant in the lung. When the lung infection causes alveolar damage, it reduces the availability of effective capillary beds in the lungs, leading to apoptosis of the megakaryocytes and resulting in thrombocytopenia [30, 31]. Although the data in the eICU-CRD predate the COVID-19 pandemic, COVID-19 resulted from infection by the severe acute respiratory syndrome coronavirus 2 (SARS-COV-2), which predominantly infects the human lungs. Therefore, it is reasonable to assume the correlation between patients with COVID-19 in previous studies and patients with viral pneumonia in the presnt study.

In addition, bacterial infections can also lead to platelet dysfunction and changes in platelet counts. Streptococcus pneumoniae is one of the leading causes of community-acquired pneumonia (CAP), and severe infection with Streptococcus pneumoniae leads to decreased platelet counts and increased mortality. One of the possible reasons is pneumolysin produced by Streptococcus pneumoniae causes apoptosis of the platelets [32].

Severe infections can lead to sepsis. Platelet counts are commonly decreased in patients with sepsis, and patients may have abnormalities in coagulation. Thirty to fifty percent of patients with sepsis have disseminated intravascular coagulation (DIC) [32–34]. DIC is a clinicopathologic syndrome that results in organ damage due to the activation of the blood clotting mechanism, which promotes fibrin deposition in the blood vessels; on the other hand, it results in the depletion of coagulation factors, such as platelets, which can lead to a bleeding tendency. These two contradictory phenomena coexist during DIC [35], and this may be related to the degree of fluctuation in the PT-INR. In this study, the proportion of patients who developed sepsis in the positive group was much greater than the proportion of those who developed sepsis in the negative group (0.27 versus 0.10).

Therefore, the lower platelet count and the more fluctuating PT-INR may indicate that the patients had sepsis and DIC due to a severe infection, which is more likely to lead to complications, including respiratory failure.

The GCS verbal score is another important predictor of respiratory failure. Alobaidy et al. reported that serum malondialdehyde (MDA) levels were statistically significantly negatively correlated with GCS scores and that total antioxidant capacity (TAC) was positively correlated with GCS scores in patients with COVID-19. SARS-CoV-2 predominantly infects the lungs of humans resulting in severe pneumonia, which implies that patients with pneumonia with low GCS scores will have higher levels of MDA and lower levels of TAC. Serum MDA is a marker of oxidative stress, and a lower GCS score means that the MDA level will be higher and the TAC level will be lower; therefore, the oxidative stress in the patient’s body will be more intense, and oxidative stress affects the severity of the disease in patients with pneumonia [30, 36]. In addition, the lower GCS verbal scores indicate a more critical situation with blurred consciousness and disrupted speech, which means that the patient’s condition is serious, with a risk of deterioration and therefore a high probability of respiratory failure.

Moreover, oxygen saturation implies the different severity of pneumonia, and patients’ severity is negatively correlated with oxygen saturation levels [37]. Therefore, the patients with pneumonia who have relatively low levels of oxygen saturation and faster respiratory rates are more likely to develop respiratory failure, even if their blood gas analysis results, such as PaO2 and PaCO2, are within the normal range. These patients need to be monitored intensively.

The data presented in Table 1 reveals that patients in the positive group had lower serum calcium levels and higher BMI than those in the negative group. Calcium ions play an important role in maintaining the excitability of the human nervous system and muscles; a decrease in calcium ion concentration may lead to respiratory muscle weakness, thereby affecting normal respiratory function. Obesity can cause various hazards. In this study, patients in the positive group had higher BMI, suggesting that obesity is an important cause of respiratory failure in patients with pneumonia. Therefore, serum calcium levels and BMI are important factors to consider for prevention of the development of respiratory failure in patients with pneumonia.

An interesting finding of this study was that the average age of patients in the positive group (62.42) was slightly lower than that of those in the negative group (67.27). A possible reason for this is that younger patients have a more active immune system and show an excessive immune response in response to lung infections, leading to cytokine storms, multiple inflammatory responses, and more severe situations [38]. However, as the patients in both positive and negative groups were older and the sample size included in this study was not large, the increased possibility of respiratory failure development in younger patients with pneumonia is not conclusive. This also emphasizes the need to pay extra attention to younger patients during actual clinical treatment to avoid underestimation of the disease progression extent due to age.

This study has some limitations. First, only 1676 eligible patients were included. An insufficient sample size could have largely affected the predictive performance of the model and cause bias in the data, thereby, reducing reliability. Second, only the eICU-CRD was used in this study, and our study lacked external validation due to the fact that many important variables such as activetx and GCS scores are unique and not recorded in other datasets. Therefore, validation based on other datasets is difficult; the predictive performance of the models in this study may be better only in the healthcare institutions included in the eICU-CRD. In the future, we would like to build machine learning models using real-world data from hospitals to improve generalization capabilities. Furhter, we need to consider the implementation of this model as an effective decision support tool in hospital information systems by collaborating with healthcare providers. Third, despite the relatively high quality of the eICU-CRD, there were many missing values for variables used in this study. Although the MICE missing value filling method was used to fill the missing variables, it may still have led to data distortion, thereby affecting the prediction performance. These issues should be addressed in future studies.

## Conclusions

In conclusion, this study used four ensemble learning algorithms, LightGBM, XGBoost, CatBoost, and random forest, to build an early prediction model for respiratory failure risk in patients with severe pneumonia. Their respective corresponding compact models were also developed to improve their utility. Among these, the CatBoost model showed the highest average AUROC on 10 different test sets and could effectively distinguish patients with pneumonia who could develop respiratory failure one hour after admission to the ICU.

## Supporting information

S1 Table Optimal hyperparameter combinations of complete models. (DOCX)

S2 Table Optimal hyperparameter combinations of compact models. (DOCX)

S1 Checklist STROBE statement—Checklist of items that should be included in reports of observational studies. (DOCX)
