# Supplementary material for: Prediction of respiratory failure risk in patients with pneumonia in the ICU using ensemble learning models
Source: PLoS One. 2023 Sep 21;18(9):e0291711. doi: 10.1371/journal.pone.0291711 (PMC10513189; doi:10.1371/journal.pone.0291711)
Supplement: S1 Table — (DOCX) [file pone.0291711.s001.docx]

**S1 Table. Optimal hyperparameter combinations of complete models**

| **Models** | **Hyperparameters** | **Values** |
| --- | --- | --- |
| LightGBM | min_split_gain | 0.10083310522615452 |
|  | subsample_freq | 1 |
|  | num_leaves | 11 |
|  | learning_rate | 0.2030117953953467 |
|  | n_estimators | 828 |
|  | max_depth | 77 |
|  | min_child_weight | 2 |
|  | min_child_samples | 32 |
|  | subsample | 0.3364809905838117 |
|  | colsample_bytree | 0.8578998043465487 |
|  | reg_alpha | 0.8876803628081088 |
|  | reg_lambda | 0.878481643066635 |
|  | max_bin | 378 |
|  | min_data_in_leaf | 51 |
|  | min_sum_hessian_in_leaf | 3.277390960331502 |
|  | bagging_fraction | 0.44041610125479524 |
|  | feature_fraction | 0.49180308519854893 |
| XGBoost | n_estimators | 536 |
|  | max_depth | 11 |
|  | learning_rate | 0.020920638463769604 |
|  | subsample | 0.765519237987702 |
|  | colsample_bytree | 0.6891660984781385 |
|  | gamma | 0.32451551334575635 |
|  | reg_alpha | 4.107307748572992 |
|  | reg_lambda | 1.0745537438252617 |
|  | min_child_weight | 1 |
|  | max_delta_step | 0.298613190322672 |
|  | scale_pos_weight | 5.792997178718542 |
| RandomForest | n_estimators | 641 |
|  | criterion | entropy |
|  | max_depth | 15 |
|  | min_samples_split | 10 |
|  | min_samples_leaf | 2 |
|  | max_features | None |
|  | bootstrap | True |
|  | class_weight | None |
| CatBoost | iterations | 865 |
|  | learning_rate | 0.04656765298643318 |
|  | depth | 6 |
|  | l2_leaf_reg | 5.230533994364776 |
|  | bagging_temperature | 0.26268757004259663 |
|  | random_strength | 0.786106471001743 |
|  | auto_class_weights | None |
