# Supplementary material for: Prediction of respiratory failure risk in patients with pneumonia in the ICU using ensemble learning models
Source: PLoS One. 2023 Sep 21;18(9):e0291711. doi: 10.1371/journal.pone.0291711 (PMC10513189; doi:10.1371/journal.pone.0291711)
Supplement: S2 Table — (DOCX) [file pone.0291711.s002.docx]

**S2 Table. Optimal hyperparameter combinations of compact models**

| **Models** | **Hyperparameters** | **Values** |
| --- | --- | --- |
| LightGBM | min_split_gain | 0.5208179066050782 |
|  | subsample_freq | 2 |
|  | num_leaves | 155 |
|  | learning_rate | 0.05262587180480516 |
|  | n_estimators | 457 |
|  | max_depth | 5 |
|  | min_child_weight | 5 |
|  | min_child_samples | 35 |
|  | subsample | 0.2542994346080063 |
|  | colsample_bytree | 0.6335070758851201 |
|  | reg_alpha | 0.7838382491839838 |
|  | reg_lambda | 0.8164194414887547 |
|  | max_bin | 678 |
|  | min_data_in_leaf | 61 |
|  | min_sum_hessian_in_leaf | 2.557810856289431 |
|  | bagging_fraction | 0.5531761467954045 |
|  | feature_fraction | 0.4142656630213855 |
| XGBoost | n_estimators | 439 |
|  | max_depth | 3 |
|  | learning_rate | 0.25639752691059176 |
|  | subsample | 0.9968661806649919 |
|  | colsample_bytree | 0.5289910918295345 |
|  | gamma | 1.0161510987952114 |
|  | reg_alpha | 0.13060573406241183 |
|  | reg_lambda | 1.5375627867253454 |
|  | min_child_weight | 1 |
|  | max_delta_step | 1.511537157203593 |
|  | scale_pos_weight | 1.350940818576408 |
| RandomForest | n_estimators | 775 |
|  | criterion | entropy |
|  | max_depth | 15 |
|  | min_samples_split | 15 |
|  | min_samples_leaf | 1 |
|  | max_features | log2 |
|  | bootstrap | True |
|  | class_weight | None |
| CatBoost | iterations | 135 |
|  | learning_rate | 0.4984635099952106 |
|  | depth | 2 |
|  | l2_leaf_reg | 9.604038708927169 |
|  | bagging_temperature | 0.4773214202375725 |
|  | random_strength | 0.7322142317698461 |
|  | auto_class_weights | None |
