# Supplementary material for: Prediction of respiratory failure risk in patients with pneumonia in the ICU using ensemble learning models
Source: PLoS One. 2023 Sep 21;18(9):e0291711. doi: 10.1371/journal.pone.0291711 (PMC10513189; doi:10.1371/journal.pone.0291711)
Supplement: S1 Checklist — (DOCX) [file pone.0291711.s003.docx]

STROBE Statement—checklist of items that should be included in reports of observational studies

|  | Item No. | Recommendation | Page  No. | Relevant text from manuscript |
| --- | --- | --- | --- | --- |
| **Title and abstract** | 1 | (*a*) Indicate the study’s design with a commonly used term in the title or the abstract | 1 | Ensemble learning prediction models for respiratory failure risk |
|  |  | (*b*) Provide in the abstract an informative and balanced summary of what was done and what was found | 2 | The CatBoost model has a significant advantage in terms of predictive performance compared to other ensemble learning models. It will help in early prediction and intervention of respiratory failure risk in patients with pneumonia in the ICU. |
| Introduction | | | |  |
| Background/rationale | 2 | Explain the scientific background and rationale for the investigation being reported | 3,4 | Respiratory failure is a common complication in patients hospitalized for pneumonia. In recent years, ensemble learning algorithms have demonstrated relatively good performances in machine learning |
| Objectives | 3 | State specific objectives, including any prespecified hypotheses | 4 | In this study, four ensemble learning algorithms, LightGBM; XGBoost; CatBoost; and random forest, were used to build early prediction models for respiratory failure risk in patients with severe pneumonia. |
| Methods | | | |  |
| Study design | 4 | Present key elements of study design early in the paper | 5-10 | Data source; study population and endpoints of interest; predictor variables; data pre-processing; complete prediction models; compact prediction models. |
| Setting | 5 | Describe the setting, locations, and relevant dates, including periods of recruitment, exposure, follow-up, and data collection | 5 | The data used in this study were obtained from the eICU Collaborative Research Database (eICU-CRD), which is a large multicenter critical care database provided by Philips Healthcare in collaboration with the MIT Computational Physiology Laboratory. It contains data on more than 200,000 critical care patients in more than 200 healthcare facilities in multiple states across the United States between 2014 and 2015. |
| Participants | 6 | (*a*) *Cohort study*—Give the eligibility criteria, and the sources and methods of selection of participants. Describe methods of follow-up  *Case-control study*—Give the eligibility criteria, and the sources and methods of case ascertainment and control selection. Give the rationale for the choice of cases and controls  *Cross-sectional study*—Give the eligibility criteria, and the sources and methods of selection of participants | 6 | patients admitted to the ICU for bacterial, viral, fungal, aspiration and parasitic pneumonia, and pneumonia labeled as other types, as per the eICU-CRD were included. For patients with multiple ICU admissions, only the records of the first ICU admission were selected. To ensure the reliability of the results, we excluded patients who did not have a diagnostic record during the period of ICU admission. Patients were not excluded based on age, as it may also influence the risk of developing respiratory failure. |
|  |  | (*b*) *Cohort study*—For matched studies, give matching criteria and number of exposed and unexposed  *Case-control study*—For matched studies, give matching criteria and the number of controls per case | 6,11 | (I) documented diagnosis of respiratory failure one hour after admission to the ICU. The textual record of the diagnoses included the following options: respiratory failure, acute respiratory failure, and ICD-9 codes 518.81, 518.84, and 518.5; (II) arterial partial pressure of oxygen (PaO2) of <60 mmHg one hour after admission to the ICU; (III) arterial partial pressure of carbon dioxide (PaCO2) >45 mmHg one hour after admission to the ICU; A total of 1676 patients with pneumonia were finally included in this study. Of these, 297 patients developed respiratory failure one hour after admission to the ICU, and the incidence of respiratory failure in patients with pneumonia in the ICU was 17.7% |
| Variables | 7 | Clearly define all outcomes, exposures, predictors, potential confounders, and effect modifiers. Give diagnostic criteria, if applicable | 7 | In this study, medical history records, laboratory results, periodic and aperiodic vital signs data, medication records, demographic data, and APACHE III calculation variables of patients were extracted before and within 1 h of ICU admission. |
| Data sources/ measurement | 8* | For each variable of interest, give sources of data and details of methods of assessment (measurement). Describe comparability of assessment methods if there is more than one group | 11-12 | The baseline characteristics of the patients with severe pneumonia are shown in Table 1 |
| Bias | 9 | Describe any efforts to address potential sources of bias | 7-8 | In this study, we did not perform outlier processing on data, such as laboratory results and vital signs, because these data may vary in real situations depending on patient conditions and certain values with special significance recorded by doctors. Therefore, if outlier processing is performed, useful features may be lost. |
| Study size | 10 | Explain how the study size was arrived at | 6 | patients admitted to the ICU for bacterial, viral, fungal, aspiration and parasitic pneumonia, and pneumonia labeled as other types, as per the eICU-CRD were included. For patients with multiple ICU admissions, only the records of the first ICU admission were selected. To ensure the reliability of the results, we excluded patients who did not have a diagnostic record during the period of ICU admission. Patients were not excluded based on age, as it may also influence the risk of developing respiratory failure. |

Continued on next page

| Quantitative variables | 11 | Explain how quantitative variables were handled in the analyses. If applicable, describe which groupings were chosen and why | 10 | Categorical variables were presented as percentages, and continuous variables were presented as mean ± standard deviation for normally distributed continuous variables or median (interquartile range) |
| --- | --- | --- | --- | --- |
| Statistical methods | 12 | (*a*) Describe all statistical methods, including those used to control for confounding | 10 | The Shapiro-Wilk test was performed to determine whether the continuous variables were normally distributed. In addition, non-normally distributed continuous variables were compared using the Mann–Whitney U test, and differences were considered statistically significant when the p-value was less than 0.001. |
|  |  | (*b*) Describe any methods used to examine subgroups and interactions | 8 | four ensemble learning algorithms (LightGBM, XGBoost, CatBoost, and random forest) were used to build prediction models for respiratory failure risk in patients with severe pneumonia in the ICU |
|  |  | (*c*) Explain how missing data were addressed | 7 | The multivariate imputations by chained equations (MICE) missing value filling method was used to fill in the missing values |
|  |  | (*d*) *Cohort study*—If applicable, explain how loss to follow-up was addressed  *Case-control study*—If applicable, explain how matching of cases and controls was addressed  *Cross-sectional study*—If applicable, describe analytical methods taking account of sampling strategy | 6,11 | The groups of patients with pneumonia who developed and did not develop respiratory failure were named the positive and negative groups, respectively.  To ensure the reliability of the results, we excluded patients who did not have a diagnostic record during the period of ICU admission. |
|  |  | (e) Describe any sensitivity analyses | N.A |  |
| Results | | | | |
| Participants | 13* | (a) Report numbers of individuals at each stage of study—eg numbers potentially eligible, examined for eligibility, confirmed eligible, included in the study, completing follow-up, and analysed | 11 | A total of 1676 patients with pneumonia were finally included in this study. |
|  |  | (b) Give reasons for non-participation at each stage | 6 | patients admitted to the ICU for bacterial, viral, fungal, aspiration and parasitic pneumonia, and pneumonia labeled as other types, as per the eICU-CRD were included. For patients with multiple ICU admissions, only the records of the first ICU admission were selected. To ensure the reliability of the results, we excluded patients who did not have a diagnostic record during the period of ICU admission. |
|  |  | (c) Consider use of a flow diagram |  |  |
| Descriptive data | 14* | (a) Give characteristics of study participants (eg demographic, clinical, social) and information on exposures and potential confounders | 11 | The baseline characteristics of the patients with severe pneumonia are shown in Table 1 |
|  |  | (b) Indicate number of participants with missing data for each variable of interest | 7 | variables with less than 80% missing values were retained to maintain the maximum amount of original patient information. |
|  |  | (c) *Cohort study*—Summarise follow-up time (eg, average and total amount) |  |  |
| Outcome data | 15* | *Cohort study*—Report numbers of outcome events or summary measures over time |  |  |
|  |  | *Case-control study—*Report numbers in each exposure category, or summary measures of exposure | 11 | A total of 1676 patients with pneumonia were finally included in this study. Of these, 297 patients developed respiratory failure one hour after admission to the ICU, and the incidence of respiratory failure in patients with pneumonia in the ICU was 17.7%. |
|  |  | *Cross-sectional study—*Report numbers of outcome events or summary measures |  |  |
| Main results | 16 | (*a*) Give unadjusted estimates and, if applicable, confounder-adjusted estimates and their precision (eg, 95% confidence interval). Make clear which confounders were adjusted for and why they were included | 13 | The average AUROC after prediction and the average accuracy at the best threshold for each complete model using the optimal combination of parameters on ten different test sets are shown in Table 2. The complete CatBoost model had the highest average AUROC after prediction on 10 different test sets (AUROC:0.866, SD:0.008) |
|  |  | (*b*) Report category boundaries when continuous variables were categorized | N.A. |  |
|  |  | (*c*) If relevant, consider translating estimates of relative risk into absolute risk for a meaningful time period |  |  |

Continued on next page

| Other analyses | 17 | Report other analyses done—eg analyses of subgroups and interactions, and sensitivity analyses | N.A |  |
| --- | --- | --- | --- | --- |
| Discussion | | | | |
| Key results | 18 | Summarise key results with reference to study objectives | 17 | Our study showed that ensemble learning algorithms can accurately predict respiratory failure risk in patients with severe pneumonia. Moreover, in this study, only laboratory results, vital signs data, medication records, APACHE III calculation variables, demographic data, and history of disease before and within one hour of admission to the ICU were used as predictor variables; therefore, the respiratory failure risk could be predicted in a relatively short period after admission of patients with pneumonia to the ICU. |
| Limitations | 19 | Discuss limitations of the study, taking into account sources of potential bias or imprecision. Discuss both direction and magnitude of any potential bias | 20 | This study has some limitations. First, only 1676 eligible patients were included. An insufficient sample size could have largely affected the predictive performance of the model and cause bias in the data, thereby, reducing reliability. Second, only the eICU-CRD was used in this study; therefore, the predictive performance of the model may be better only in the healthcare institutions included in this database. Third, despite the relatively high quality of the eICU-CRD, there were many missing values for variables used in this study. |
| Interpretation | 20 | Give a cautious overall interpretation of results considering objectives, limitations, multiplicity of analyses, results from similar studies, and other relevant evidence | 20 | In conclusion, this study used four ensemble learning algorithms, LightGBM, XGBoost, CatBoost, and random forest, to build an early prediction model for respiratory failure risk in patients with severe pneumonia. Their respective corresponding compact models were also developed to improve their utility. Among these, the CatBoost model showed the strongest predictive performance and could effectively distinguish patients with pneumonia who could develop respiratory failure one hour after admission to the ICU. |
| Generalisability | 21 | Discuss the generalisability (external validity) of the study results | 20 | only the eICU-CRD was used in this study; therefore, the predictive performance of the model may be better only in the healthcare institutions included in this database |
| Other information | |  | | |
| Funding | 22 | Give the source of funding and the role of the funders for the present study and, if applicable, for the original study on which the present article is based | N.A. |  |

*Give information separately for cases and controls in case-control studies and, if applicable, for exposed and unexposed groups in cohort and cross-sectional studies.

**Note:** An Explanation and Elaboration article discusses each checklist item and gives methodological background and published examples of transparent reporting. The STROBE checklist is best used in conjunction with this article (freely available on the Web sites of PLoS Medicine at http://www.plosmedicine.org/, Annals of Internal Medicine at http://www.annals.org/, and Epidemiology at http://www.epidem.com/). Information on the STROBE Initiative is available at www.strobe-statement.org.
